# 1-Acetyl-*t*-3-ethyl-*r*-2,*c*-6-bis­(4-methoxy­phen­yl)piperidin-4-one

**DOI:** 10.1107/S1600536809054737

**Published:** 2010-01-09

**Authors:** K. Ravichandran, S. Ponnuswamy, R. Rajesh, V. Mohanraj, M. N. Ponnuswamy

**Affiliations:** a Centre of Advanced Study in Crystallography and Biophysics, University of Madras, Guindy Campus, Chennai 600 025, India; b Department of Chemistry, Government Arts College (Autonomous), Coimbatore 641 018, India

## Abstract

In the title compound, C_23_H_27_NO_4_, the piperidine ring adopts a distorted boat conformation. The meth­oxy groups lie in the plane of benzene rings to which they are attached [maximum deviations of 0.014 (3) and 0.007 (3) Å]. The benzene rings are oriented at angles of 67.2 (1) and 87.0 (1)° with respect to the best plane through the four co-planar atoms of the piperidine ring.

## Related literature

For general background to piperidine derivatives, see: Aridoss *et al.* (2008 ▶). For asymmetry parameters, see: Nardelli (1983 ▶). For puckering parameters, see: Cremer & Pople (1975 ▶).
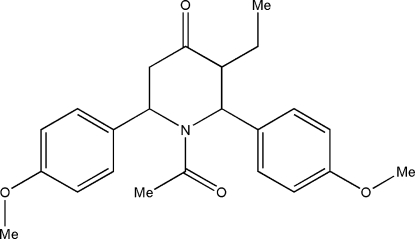


## Experimental

### 

#### Crystal data

C_23_H_27_NO_4_
*M* *_r_* = 381.46
Orthorhombic, 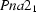

*a* = 8.6736 (11) Å
*b* = 13.4578 (16) Å
*c* = 17.547 (2) Å
*V* = 2048.2 (4) Å^3^
*Z* = 4
Mo *K*α radiation
μ = 0.08 mm^−1^
*T* = 293 K
0.25 × 0.23 × 0.20 mm

#### Data collection

Bruker SMART APEXII area-detector diffractometer
Absorption correction: multi-scan (*SADABS*; Bruker, 2008 ▶) *T*_min_ = 0.979, *T*_max_ = 0.983
11102 measured reflections
2646 independent reflections
1926 reflections with *I* > 2σ(*I*)
*R*_int_ = 0.036

#### Refinement

*R*[*F* ^2^ > 2σ(*F* ^2^)] = 0.046
*wR*(*F* ^2^) = 0.138
*S* = 1.08
2646 reflections
257 parameters
1 restraint
H-atom parameters constrained
Δρ_max_ = 0.42 e Å^−3^
Δρ_min_ = −0.19 e Å^−3^

### 

Data collection: *APEX2* (Bruker, 2008 ▶); cell refinement: *SAINT* (Bruker, 2008 ▶); data reduction: *SAINT*; program(s) used to solve structure: *SHELXS97* (Sheldrick, 2008 ▶); program(s) used to refine structure: *SHELXL97* (Sheldrick, 2008 ▶); molecular graphics: *ORTEP-3* (Farrugia, 1997 ▶); software used to prepare material for publication: *SHELXL97* and *PLATON* (Spek, 2009 ▶).

## Supplementary Material

Crystal structure: contains datablocks global, I. DOI: 10.1107/S1600536809054737/bt5144sup1.cif

Structure factors: contains datablocks I. DOI: 10.1107/S1600536809054737/bt5144Isup2.hkl

Additional supplementary materials:  crystallographic information; 3D view; checkCIF report

## Figures and Tables

**Fig. 1.**
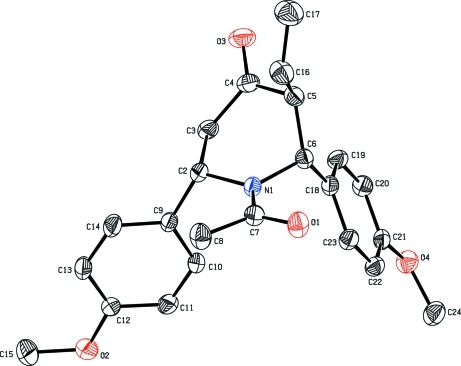
Perspective view of the molecule showing the thermal ellipsoids are drawn at 30% probability level. H atoms have been omitted for clarity.

**Fig. 2.**
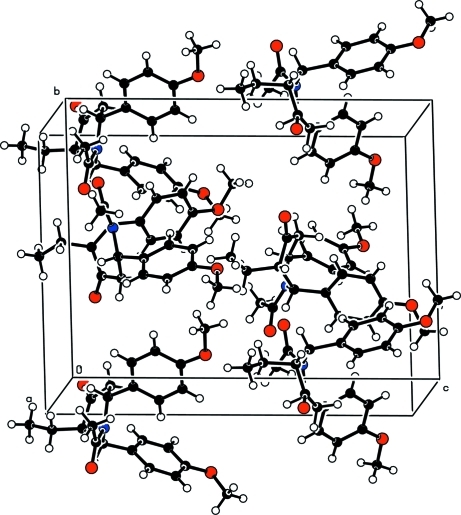
The crystal packing of the molecules viewed down a–axis.

## supplementary crystallographic information

### Comment

Piperidine derivatives are the valued heterocyclic compounds in the field of medicinal chemistry. The compounds possessing an amide bond linkage have a wide range of biological activities such as antimicrobial, antinflammatory, antiviral, antimalarial and general anaesthetics. Furtheremore, the amides derived from chloroacetylchloride also gain significant importance in medicinal field as evidenced by their varied pharmacological activities (Aridoss *et al.*,  2008). The crystallographic study of the title compound has been carried out to establish the molecular structur

The *ORTEP* diagram of the title compound is shown in Fig. 1. The piperidine ring in the molecule adopts a distorted boat conformation. The puckering parameters (Cremer & Pople, 1975) and the asymmetry parameters (Nardelli, 1983) are: q_2_ = 0.636 (3) Å, q_3_ = 0.089 (3) Å, φ_2_ = 286.2 (2)° and Δ_s_(C3 & C6)= 15.4 (3)°. The methoxy groups lie in the plane of phenyl rings and the phenyl rings are oriented at angles of 67.2 (1)° and 87.0(1°) with the best plane of piperidine ring. The sum of the bond angles around the atom N1(358.5°) of the piperidine ring in the molecule is in accordance with *sp*^2^ hybridization. The crystal structure is stabilized by intramolecular  C—H···O interactions.

### Experimental

To a solution of t-3-ethyl-r-2,c-6-bis(4-methoxyphenyl)piperidin-4-one (3.39 g) in anhydrous benzene (60 ml) was added triethylamine (2.78) and acetylchloride (1.42 ml). The contents were allowed to reflux on a water bath for 12 h. The precipitated ammonium salt was filtered off and the filterate was washed with water. The organic layer was dried over anhydrous Na_2_SO_4_, concentrated and crystallized from benzene:pet-ether (60–80°C) in the ratio of 9:1.

### Refinement

In the absense of anomalous scatterers Friedel pairs were merged. The C bound H atoms positioned geometrically (C—H=0.93–0.98 Å) and allowed to ride on their parent atoms, with 1.5*U*_eq_(C) for methyl H and 1.2 *U*_eq_(C) for other H atoms.

### Crystal data

**Table:**

C_23_H_27_NO_4_	*F*(000) = 816
*M**_r_* = 381.46	*D*_x_ = 1.237 Mg m^−^^3^
Orthorhombic, *P**n**a*2_1_	Mo *K*α radiation, λ = 0.71073 Å
Hall symbol: P 2c -2n	Cell parameters from 1987 reflections
*a* = 8.6736 (11) Å	θ = 1.9–28.5°
*b* = 13.4578 (16) Å	µ = 0.08 mm^−^^1^
*c* = 17.547 (2) Å	*T* = 293 K
*V* = 2048.2 (4) Å^3^	Block, colorless
*Z* = 4	0.25 × 0.23 × 0.20 mm

### Data collection

**Table:**

Bruker SMART APEXII area-detector diffractometer	2646 independent reflections
Radiation source: fine-focus sealed tube	1926 reflections with *I* > 2σ(*I*)
graphite	*R*_int_ = 0.036
ω and φ scans	θ_max_ = 28.5°, θ_min_ = 1.9°
Absorption correction: multi-scan (*SADABS*; Bruker, 2008)	*h* = −11→8
*T*_min_ = 0.979, *T*_max_ = 0.983	*k* = −17→13
11102 measured reflections	*l* = −16→23

### Refinement

**Table:**

Refinement on *F*^2^	Primary atom site location: structure-invariant direct methods
Least-squares matrix: full	Secondary atom site location: difference Fourier map
*R*[*F*^2^ > 2σ(*F*^2^)] = 0.046	Hydrogen site location: inferred from neighbouring sites
*wR*(*F*^2^) = 0.138	H-atom parameters constrained
*S* = 1.08	*w* = 1/[σ^2^(*F*_o_^2^) + (0.077*P*)^2^ + 0.0634*P*] where *P* = (*F*_o_^2^ + 2*F*_c_^2^)/3
2646 reflections	(Δ/σ)_max_ < 0.001
257 parameters	Δρ_max_ = 0.42 e Å^−^^3^
1 restraint	Δρ_min_ = −0.19 e Å^−^^3^

### Special details

**Table:**

Geometry. All e.s.d.'s (except the e.s.d. in the dihedral angle between two l.s. planes) are estimated using the full covariance matrix. The cell e.s.d.'s are taken into account individually in the estimation of e.s.d.'s in distances, angles and torsion angles; correlations between e.s.d.'s in cell parameters are only used when they are defined by crystal symmetry. An approximate (isotropic) treatment of cell e.s.d.'s is used for estimating e.s.d.'s involving l.s. planes.
Refinement. Refinement of *F*^2^ against ALL reflections. The weighted *R*-factor *wR* and goodness of fit *S* are based on *F*^2^, conventional *R*-factors *R* are based on *F*, with *F* set to zero for negative *F*^2^. The threshold expression of *F*^2^ > σ(*F*^2^) is used only for calculating *R*-factors(gt) *etc*. and is not relevant to the choice of reflections for refinement. *R*-factors based on *F*^2^ are statistically about twice as large as those based on *F*, and *R*- factors based on ALL data will be even larger.

### Fractional atomic coordinates and isotropic or equivalent isotropic displacement parameters (Å^2^)

**Table:**

	*x*	*y*	*z*	*U*_iso_*/*U*_eq_	
O1	0.2257 (3)	0.72781 (16)	0.10022 (18)	0.0773 (7)	
O2	−0.1555 (3)	0.37114 (19)	0.38952 (16)	0.0789 (7)	
O3	0.6511 (3)	0.3748 (2)	0.11657 (17)	0.0849 (8)	
O4	0.6382 (3)	0.68839 (18)	0.45689 (13)	0.0698 (6)	
N1	0.2969 (3)	0.57593 (15)	0.14275 (14)	0.0496 (5)	
C2	0.2612 (3)	0.4685 (2)	0.15230 (17)	0.0519 (6)	
H2	0.2212	0.4437	0.1037	0.062*	
C3	0.4078 (4)	0.4093 (2)	0.1716 (2)	0.0607 (8)	
H3A	0.4284	0.4169	0.2256	0.073*	
H3B	0.3870	0.3395	0.1624	0.073*	
C4	0.5522 (4)	0.4374 (2)	0.12812 (19)	0.0633 (8)	
C5	0.5648 (3)	0.5426 (2)	0.10367 (18)	0.0591 (7)	
H5	0.6716	0.5643	0.1113	0.071*	
C6	0.4594 (3)	0.6100 (2)	0.15094 (17)	0.0517 (6)	
H6	0.4638	0.6754	0.1265	0.062*	
C7	0.1935 (3)	0.6403 (2)	0.11167 (19)	0.0578 (7)	
C8	0.0345 (4)	0.6026 (3)	0.0908 (3)	0.0780 (10)	
H8A	−0.0210	0.6538	0.0644	0.117*	
H8B	−0.0202	0.5847	0.1364	0.117*	
H8C	0.0440	0.5454	0.0585	0.117*	
C9	0.1414 (3)	0.4477 (2)	0.21308 (17)	0.0506 (6)	
C10	0.1417 (3)	0.4952 (2)	0.28316 (19)	0.0597 (7)	
H10	0.2113	0.5465	0.2919	0.072*	
C11	0.0419 (4)	0.4682 (3)	0.3394 (2)	0.0655 (8)	
H11	0.0451	0.5012	0.3859	0.079*	
C12	−0.0635 (4)	0.3930 (2)	0.32890 (18)	0.0550 (7)	
C13	−0.0700 (4)	0.3475 (3)	0.2595 (2)	0.0731 (10)	
H13	−0.1435	0.2987	0.2503	0.088*	
C14	0.0336 (5)	0.3744 (3)	0.2025 (2)	0.0738 (10)	
H14	0.0296	0.3417	0.1558	0.089*	
C15	−0.2642 (7)	0.2920 (4)	0.3811 (4)	0.120 (2)	
H15A	−0.3393	0.3098	0.3433	0.181*	
H15B	−0.3149	0.2804	0.4289	0.181*	
H15C	−0.2113	0.2328	0.3655	0.181*	
C16	0.5258 (5)	0.5529 (3)	0.0179 (2)	0.0792 (10)	
H16A	0.4326	0.5154	0.0076	0.095*	
H16B	0.5041	0.6221	0.0071	0.095*	
C17	0.6463 (6)	0.5190 (5)	−0.0336 (3)	0.1079 (16)	
H17A	0.7329	0.5632	−0.0306	0.162*	
H17B	0.6075	0.5182	−0.0848	0.162*	
H17C	0.6780	0.4532	−0.0194	0.162*	
C18	0.5072 (3)	0.62705 (19)	0.23351 (17)	0.0509 (6)	
C19	0.6239 (3)	0.5761 (2)	0.27049 (19)	0.0586 (7)	
H19	0.6770	0.5265	0.2445	0.070*	
C20	0.6632 (3)	0.5970 (2)	0.34454 (19)	0.0580 (7)	
H20	0.7408	0.5608	0.3683	0.070*	
C21	0.5885 (3)	0.6715 (2)	0.38383 (18)	0.0541 (7)	
C22	0.4715 (4)	0.7235 (2)	0.3481 (2)	0.0611 (8)	
H22	0.4199	0.7738	0.3739	0.073*	
C23	0.4317 (3)	0.7002 (2)	0.2739 (2)	0.0583 (7)	
H23	0.3518	0.7349	0.2506	0.070*	
C24	0.5793 (5)	0.7729 (3)	0.4950 (2)	0.0799 (10)	
H24A	0.5958	0.8309	0.4641	0.120*	
H24B	0.6314	0.7809	0.5429	0.120*	
H24C	0.4709	0.7644	0.5037	0.120*	

### Atomic displacement parameters (Å^2^)

**Table:**

	*U*^11^	*U*^22^	*U*^33^	*U*^12^	*U*^13^	*U*^23^
O1	0.0703 (13)	0.0523 (12)	0.1093 (19)	0.0031 (10)	−0.0158 (14)	0.0160 (12)
O2	0.0801 (16)	0.0746 (16)	0.0820 (16)	−0.0055 (12)	0.0234 (13)	−0.0043 (13)
O3	0.0738 (15)	0.0847 (17)	0.0962 (19)	0.0267 (13)	0.0087 (14)	−0.0157 (15)
O4	0.0709 (14)	0.0749 (15)	0.0637 (13)	−0.0019 (11)	−0.0078 (12)	−0.0087 (11)
N1	0.0480 (12)	0.0419 (11)	0.0591 (13)	−0.0018 (9)	−0.0053 (10)	−0.0002 (10)
C2	0.0573 (15)	0.0446 (14)	0.0538 (14)	−0.0033 (12)	−0.0011 (13)	−0.0076 (12)
C3	0.0660 (18)	0.0446 (15)	0.0714 (19)	0.0091 (13)	0.0066 (15)	−0.0021 (14)
C4	0.0606 (17)	0.0687 (19)	0.0606 (17)	0.0108 (15)	0.0008 (14)	−0.0132 (15)
C5	0.0490 (15)	0.0695 (18)	0.0588 (16)	−0.0026 (13)	0.0018 (13)	−0.0014 (15)
C6	0.0467 (14)	0.0494 (14)	0.0590 (15)	−0.0025 (11)	−0.0046 (12)	0.0019 (13)
C7	0.0560 (16)	0.0556 (16)	0.0620 (17)	0.0023 (13)	−0.0056 (14)	0.0024 (14)
C8	0.0608 (19)	0.071 (2)	0.102 (3)	−0.0010 (16)	−0.0243 (19)	0.008 (2)
C9	0.0516 (14)	0.0403 (13)	0.0599 (16)	−0.0020 (11)	−0.0060 (12)	−0.0043 (12)
C10	0.0532 (15)	0.0583 (17)	0.0677 (18)	−0.0134 (13)	0.0010 (14)	−0.0164 (15)
C11	0.0628 (18)	0.070 (2)	0.0634 (18)	−0.0034 (15)	−0.0004 (15)	−0.0217 (16)
C12	0.0527 (15)	0.0481 (15)	0.0642 (18)	0.0015 (12)	0.0054 (13)	−0.0045 (13)
C13	0.080 (2)	0.0620 (19)	0.077 (2)	−0.0282 (17)	0.0116 (18)	−0.0147 (17)
C14	0.088 (2)	0.070 (2)	0.0634 (18)	−0.0273 (18)	0.0077 (18)	−0.0215 (17)
C15	0.120 (4)	0.100 (3)	0.141 (4)	−0.048 (3)	0.058 (4)	−0.017 (3)
C16	0.076 (2)	0.101 (3)	0.0604 (18)	−0.010 (2)	0.0017 (17)	0.004 (2)
C17	0.103 (3)	0.139 (5)	0.082 (3)	−0.006 (3)	0.011 (3)	−0.012 (3)
C18	0.0487 (13)	0.0449 (14)	0.0591 (15)	−0.0048 (12)	−0.0038 (12)	−0.0002 (12)
C19	0.0541 (15)	0.0527 (16)	0.0690 (19)	0.0091 (13)	−0.0027 (14)	−0.0082 (14)
C20	0.0498 (15)	0.0583 (17)	0.0658 (18)	0.0051 (12)	−0.0100 (14)	0.0008 (14)
C21	0.0489 (13)	0.0492 (16)	0.0642 (17)	−0.0092 (12)	−0.0038 (13)	−0.0049 (13)
C22	0.0526 (15)	0.0519 (16)	0.079 (2)	0.0045 (12)	−0.0048 (15)	−0.0137 (15)
C23	0.0527 (14)	0.0508 (15)	0.0715 (19)	0.0069 (12)	−0.0114 (14)	−0.0053 (14)
C24	0.091 (2)	0.076 (2)	0.073 (2)	−0.0085 (19)	−0.006 (2)	−0.0173 (19)

### Geometric parameters (Å, °)

**Table:**

O1—C7	1.227 (4)	C11—C12	1.376 (5)
O2—C12	1.362 (4)	C11—H11	0.9300
O2—C15	1.430 (5)	C12—C13	1.364 (5)
O3—C4	1.219 (4)	C13—C14	1.393 (5)
O4—C21	1.372 (4)	C13—H13	0.9300
O4—C24	1.415 (5)	C14—H14	0.9300
N1—C7	1.361 (4)	C15—H15A	0.9600
N1—C2	1.488 (4)	C15—H15B	0.9600
N1—C6	1.489 (3)	C15—H15C	0.9600
C2—C9	1.515 (4)	C16—C17	1.456 (6)
C2—C3	1.538 (4)	C16—H16A	0.9700
C2—H2	0.9800	C16—H16B	0.9700
C3—C4	1.514 (5)	C17—H17A	0.9600
C3—H3A	0.9700	C17—H17B	0.9600
C3—H3B	0.9700	C17—H17C	0.9600
C4—C5	1.484 (5)	C18—C23	1.379 (4)
C5—C6	1.532 (4)	C18—C19	1.384 (4)
C5—C16	1.548 (5)	C19—C20	1.373 (5)
C5—H5	0.9800	C19—H19	0.9300
C6—C18	1.525 (4)	C20—C21	1.378 (4)
C6—H6	0.9800	C20—H20	0.9300
C7—C8	1.514 (4)	C21—C22	1.382 (4)
C8—H8A	0.9600	C22—C23	1.383 (5)
C8—H8B	0.9600	C22—H22	0.9300
C8—H8C	0.9600	C23—H23	0.9300
C9—C14	1.371 (4)	C24—H24A	0.9600
C9—C10	1.386 (4)	C24—H24B	0.9600
C10—C11	1.362 (5)	C24—H24C	0.9600
C10—H10	0.9300		
			
C12—O2—C15	117.8 (3)	C13—C12—C11	118.5 (3)
C21—O4—C24	117.5 (3)	O2—C12—C11	116.3 (3)
C7—N1—C2	121.7 (2)	C12—C13—C14	119.9 (3)
C7—N1—C6	117.8 (2)	C12—C13—H13	120.1
C2—N1—C6	119.0 (2)	C14—C13—H13	120.1
N1—C2—C9	113.7 (2)	C9—C14—C13	121.9 (3)
N1—C2—C3	110.9 (2)	C9—C14—H14	119.0
C9—C2—C3	108.5 (2)	C13—C14—H14	119.0
N1—C2—H2	107.9	O2—C15—H15A	109.5
C9—C2—H2	107.9	O2—C15—H15B	109.5
C3—C2—H2	107.9	H15A—C15—H15B	109.5
C4—C3—C2	116.3 (3)	O2—C15—H15C	109.5
C4—C3—H3A	108.2	H15A—C15—H15C	109.5
C2—C3—H3A	108.2	H15B—C15—H15C	109.5
C4—C3—H3B	108.2	C17—C16—C5	114.8 (4)
C2—C3—H3B	108.2	C17—C16—H16A	108.6
H3A—C3—H3B	107.4	C5—C16—H16A	108.6
O3—C4—C5	124.0 (3)	C17—C16—H16B	108.6
O3—C4—C3	119.6 (3)	C5—C16—H16B	108.6
C5—C4—C3	116.4 (3)	H16A—C16—H16B	107.5
C4—C5—C6	111.4 (3)	C16—C17—H17A	109.5
C4—C5—C16	110.5 (3)	C16—C17—H17B	109.5
C6—C5—C16	110.1 (3)	H17A—C17—H17B	109.5
C4—C5—H5	108.3	C16—C17—H17C	109.5
C6—C5—H5	108.3	H17A—C17—H17C	109.5
C16—C5—H5	108.3	H17B—C17—H17C	109.5
N1—C6—C18	113.3 (2)	C23—C18—C19	117.4 (3)
N1—C6—C5	109.3 (2)	C23—C18—C6	117.8 (3)
C18—C6—C5	116.2 (2)	C19—C18—C6	124.7 (3)
N1—C6—H6	105.7	C20—C19—C18	121.6 (3)
C18—C6—H6	105.7	C20—C19—H19	119.2
C5—C6—H6	105.7	C18—C19—H19	119.2
O1—C7—N1	121.8 (3)	C19—C20—C21	120.4 (3)
O1—C7—C8	119.3 (3)	C19—C20—H20	119.8
N1—C7—C8	118.9 (3)	C21—C20—H20	119.8
C7—C8—H8A	109.5	O4—C21—C20	116.1 (3)
C7—C8—H8B	109.5	O4—C21—C22	124.8 (3)
H8A—C8—H8B	109.5	C20—C21—C22	119.1 (3)
C7—C8—H8C	109.5	C21—C22—C23	119.7 (3)
H8A—C8—H8C	109.5	C21—C22—H22	120.2
H8B—C8—H8C	109.5	C23—C22—H22	120.2
C14—C9—C10	117.0 (3)	C22—C23—C18	121.8 (3)
C14—C9—C2	120.3 (3)	C22—C23—H23	119.1
C10—C9—C2	122.5 (2)	C18—C23—H23	119.1
C11—C10—C9	121.2 (3)	O4—C24—H24A	109.5
C11—C10—H10	119.4	O4—C24—H24B	109.5
C9—C10—H10	119.4	H24A—C24—H24B	109.5
C10—C11—C12	121.4 (3)	O4—C24—H24C	109.5
C10—C11—H11	119.3	H24A—C24—H24C	109.5
C12—C11—H11	119.3	H24B—C24—H24C	109.5
C13—C12—O2	125.2 (3)		
			
C7—N1—C2—C9	−70.2 (3)	C2—C9—C10—C11	−173.5 (3)
C6—N1—C2—C9	123.6 (3)	C9—C10—C11—C12	−0.3 (5)
C7—N1—C2—C3	167.3 (3)	C15—O2—C12—C13	2.3 (6)
C6—N1—C2—C3	1.1 (4)	C15—O2—C12—C11	−178.7 (4)
N1—C2—C3—C4	−41.2 (4)	C10—C11—C12—C13	−2.1 (5)
C9—C2—C3—C4	−166.7 (3)	C10—C11—C12—O2	178.8 (3)
C2—C3—C4—O3	−151.4 (3)	O2—C12—C13—C14	−178.1 (3)
C2—C3—C4—C5	30.1 (4)	C11—C12—C13—C14	3.0 (6)
O3—C4—C5—C6	−157.9 (3)	C10—C9—C14—C13	−0.8 (6)
C3—C4—C5—C6	20.6 (4)	C2—C9—C14—C13	174.5 (3)
O3—C4—C5—C16	79.5 (4)	C12—C13—C14—C9	−1.5 (7)
C3—C4—C5—C16	−102.0 (3)	C4—C5—C16—C17	−75.5 (5)
C7—N1—C6—C18	110.1 (3)	C6—C5—C16—C17	161.1 (4)
C2—N1—C6—C18	−83.1 (3)	N1—C6—C18—C23	−63.3 (3)
C7—N1—C6—C5	−118.6 (3)	C5—C6—C18—C23	168.9 (3)
C2—N1—C6—C5	48.2 (3)	N1—C6—C18—C19	118.4 (3)
C4—C5—C6—N1	−58.7 (3)	C5—C6—C18—C19	−9.4 (4)
C16—C5—C6—N1	64.1 (3)	C23—C18—C19—C20	0.2 (5)
C4—C5—C6—C18	71.0 (3)	C6—C18—C19—C20	178.4 (3)
C16—C5—C6—C18	−166.1 (3)	C18—C19—C20—C21	−1.2 (5)
C2—N1—C7—O1	−176.2 (3)	C24—O4—C21—C20	171.3 (3)
C6—N1—C7—O1	−9.9 (5)	C24—O4—C21—C22	−8.4 (4)
C2—N1—C7—C8	3.8 (5)	C19—C20—C21—O4	−178.5 (3)
C6—N1—C7—C8	170.2 (3)	C19—C20—C21—C22	1.2 (5)
N1—C2—C9—C14	142.0 (3)	O4—C21—C22—C23	179.6 (3)
C3—C2—C9—C14	−94.2 (4)	C20—C21—C22—C23	−0.1 (5)
N1—C2—C9—C10	−43.0 (4)	C21—C22—C23—C18	−1.0 (5)
C3—C2—C9—C10	80.8 (3)	C19—C18—C23—C22	0.9 (5)
C14—C9—C10—C11	1.7 (5)	C6—C18—C23—C22	−177.4 (3)

### Hydrogen-bond geometry (Å, °)

**Table:**

*D*—H···*A*	*D*—H	H···*A*	*D*···*A*	*D*—H···*A*
C6—H6···O1	0.98	2.23	2.723 (3)	110
